# National Association of Dental Faculties

**Published:** 1900-05

**Authors:** 


### NATIONAL ASSOCIATION OF DENTAL FACULTIES.

THE official announcement of the meeting of this body will be found upon another page. It is to be regretted that any change has been made from the old and long-established plan of meeting in advance of the National Dental Association. It is feared it will not be in the interest of either of these two important organizations. It would seem a judicious move to change to the original method of meeting in advance of the National organization. If this is not done, the latter association will suffer in the non-attendance of the men most needed to give it vitality.

